# How can artificial intelligence optimize value-based contracting?

**DOI:** 10.1186/s40545-022-00475-3

**Published:** 2022-11-18

**Authors:** Jose Luis Poveda, Rosa Bretón-Romero, Carlos Del Rio-Bermudez, Miren Taberna, Ignacio H. Medrano

**Affiliations:** 1 Pharmacy Department, Drug Clinical Area, University and Polytechnic Hospital La Fe, Avda. Fernando Abril Martorell 106, 46026 Valencia, Spain; 2 Savana Research SL, Madrid, Spain; 3 MedSavana SL, Madrid, Spain

## Abstract

Efforts in the pharmaceutical market have been aimed at ensuring that the benefits obtained from the introduction of new therapies justify the associated costs. In recent years, drug payment models in healthcare have undergone a dramatic shift from focusing on volume (i.e., size of the target clinical population) to focusing on value (i.e., drug performance in real-world settings). In this context, value-based contracts (VBCs) were designed to align the payment of a drug to its clinical performance outside clinical trials by evaluating the effectiveness using real-word evidence (RWE). Despite their widespread implementation, different factors jeopardize the application of VBCs to most marketed drugs in a near future, including the need for easily measurable and relevant outcomes associated with clinical improvements, and access to a large patient population to assess said outcomes. Here, we argue that the extraction and analysis of massive amounts of RWE captured in patients’ electronic health records (EHRs) will circumvent these issues and optimize negotiations in VBCs. Particularly, the use of Natural Language Processing (NLP) has proven successful in the analysis of structured and unstructured clinical information in EHRs in multicenter research studies. Thus, the application of NLP to analyze patient-centered information in EHRs in the context of innovative contracting can be utterly beneficial as it enables the real-time evaluation of treatment response and financial impact in real-world settings.

**Supplementary Information:**

The online version contains supplementary material available at 10.1186/s40545-022-00475-3.

## Introduction

### Innovative contracting in pharmaceutical markets: value-based contracts

The evaluation of the benefits associated with a given drug and price negotiations have been traditionally focused on the size of the target population, health system goals, and potential clinical outcomes [1]. These fixed price policies disregard the use and drug-effectiveness in real-world; while the payer assumes the entire risk in terms of budgetary conditions and health impact in the clinical practice, the manufacturer deals with the uncertain profitability of the investment [1]. During the last two decades, different strategies in the pharmaceutical market have aimed to ensure that the benefit obtained from the introduction of new therapies justifies the associated costs, leading to a tight collaboration between payers and manufacturers to design innovative contractual agreements [2, 3].

Innovative contracts are payment arrangements in the pharmaceutical market outside traditional fixed-cost-per-unit and rebating practices [4]. Manufacturers are incentivized to engage in these contracts to differentiate their drug from an established competitor, or to establish its clinical value in a space, where it has not been fully demonstrated [2]. Moreover, payers can reduce the risk associated with the uncertain performance of new therapies by taking into account real-world data (RWD) [2, 5]. Value-based contracts (VBCs) (also known as risk-sharing or pay-for-performance agreements) encompass different innovative contracting strategies that do not fix payment rates on volume, but rather on the achievement of specific therapeutic goal, also improve patient access to novel (but often costly) therapies [3]. Thus, VBCs are designed to align the payment of a drug to its real-world performance based on clinical outcomes previously defined by the stakeholders (manufacturers and payers) [2, 3, 6, 7]. VBCs are more appropriate under certain circumstances, including evaluation of expensive products that are a priority for payers, differentiation of a product from an established competitor, products with unproven effectiveness, products that will cover a therapeutic gap, and products targeting a small patient population [2, 4, 6].

Although the implementation of VBCs worldwide has exponentially increased in recent years, only a small number of these have been reported publicly [2, 8]. A list of publicly available contracts in the United States, European Union (Spain and Italy), and the United Kingdom in the past 5 years is shown in the Additional file 1.

In the United States, the shift between volume-fixed prices to value-based agreements began in the 00 s, influenced by the Affordable Care Act and reinforced by the Medicare Access and CHIP Reauthorization Act of 2015 [8]. However, it is difficult to obtain a representative picture of performance-based agreements due to the high number of payers and lack of transparency in the private payer sector [9].

In the European Union, VBCs are also known as Managed Entry Agreements (MEAs) and include finance-based agreements and performance-based agreements [10]. VBCs have become established approaches to balance budgetary pressures on healthcare systems since the 1900s, but their implementation varies geographically [4, 6]. The experience with outcome-based contracts (OBCs) is longer and more prolific in Italy, where MEAs represented about 35% of all publicly disclosed contracts and were required for all drugs in oncology, immunology, and some rare diseases in 2017 [4, 11]. In Spain, the implementation of these agreements is generally performed at the hospital level, although they can involve regional and even national negotiations [12, 13]. In 2018, the first pay-per-performance agreement publicly approved in Spain opened the doors for the Therapeutic Value of Medicines or the Valtermed initiatives, and enabled the implementation of VBCs at the national level [14, 15].

In the United Kingdom, the National Institute for Health and Clinical Excellence (NICE) is responsible for assessing the clinical and cost-effectiveness of medicines, and the National Health Service is legally obliged to provide funding for all treatments recommended by NICE [16]. In this context, the first OBCs were awarded in 2011 and have grown substantially since then [17].

### Challenges in the use of real-world evidence (RWE) to guide VBC

Different factors jeopardize the application of VBCs to most marketed drugs in a near future, including the need for measurable and clinically relevant outcomes associated with financial and/or clinical improvements, the risk sharing between the manufacturer and the payer, and the need for a sufficiently size patient population [5, 8, 18]. Thus, stakeholders must identify, extract, and analyze data to validate whether the desirable outcomes are achieved or not in real-world settings [8].

Potential sources of RWD include electronic prescribing systems, insurance claims, and electronic health records (EHRs) [8, 19]. EHRs capture patient-centered information including medical history, diagnosis, treatment outcomes, adverse drug reactions, prescriptions, genome sequencing, and laboratory testing, among others [20–22]. This information, which reflects actual clinical practice, is stored longitudinally for each patient and is easily scalable [23]. Thus, physician´s notes, referring letters, specialist´s reports, discharge information, and summaries of communications between doctors and patients jotted down by health professionals are critical for the evaluation of the treatment and optimization of hospital resources real-world settings. However, RWD from EHR has been an underused resource due to its complexity [19, 24].

In response to this need, artificial intelligence (AI), and in particular the natural language processing (NLP) tools, have been developed to extract, organize, and interpret large amounts of data regardless their linguistic complexity, enabling the use of unstructured text within the EHRs in a non-time-consuming and inexpensive manner [23, 25]. Research applications of NLP using EHRs offer insightful descriptive and predictive results into clinical populations, patient management, pharmacovigilance, and computerized clinical decision support, among others [26–30]. Furthermore, information extracted from the EHRs using NLP can be used for eligibility analysis and reduction of population heterogeneity during clinical trial recruitment, or lead to the selection of those patients with a higher probability of having a measurable clinical endpoint [31]. Although validation of NLP systems in clinical settings guarantee optimal precision and sensitivity in the extraction of all desired outcomes while securing patients’ privacy via the anonymization of EHRs, some challenges of the method have to be considered, including the detection of temporal relationships and causal inferences, understanding of homonyms and acronyms, and identification of negated terms [32], [33]. Importantly, the quality and validity of the results yielded by NLP relied on the completeness and accuracy of EHRs. Thus, raising awareness among healthcare professionals about the importance of EHRs completeness would boost the quality of hospital resource management [23].

### How could AI and NLP assist negotiations and outcome evaluations in VBCs?

Despite key information regarding effectiveness and safety of marketed drugs is available in patients’ EHRs, and there is a growing recognition of the value of using RWE for regulatory decision-making [34], the use of this information in the design and evaluation of VBCs is virtually nonexistent.

**Fig. 1:**
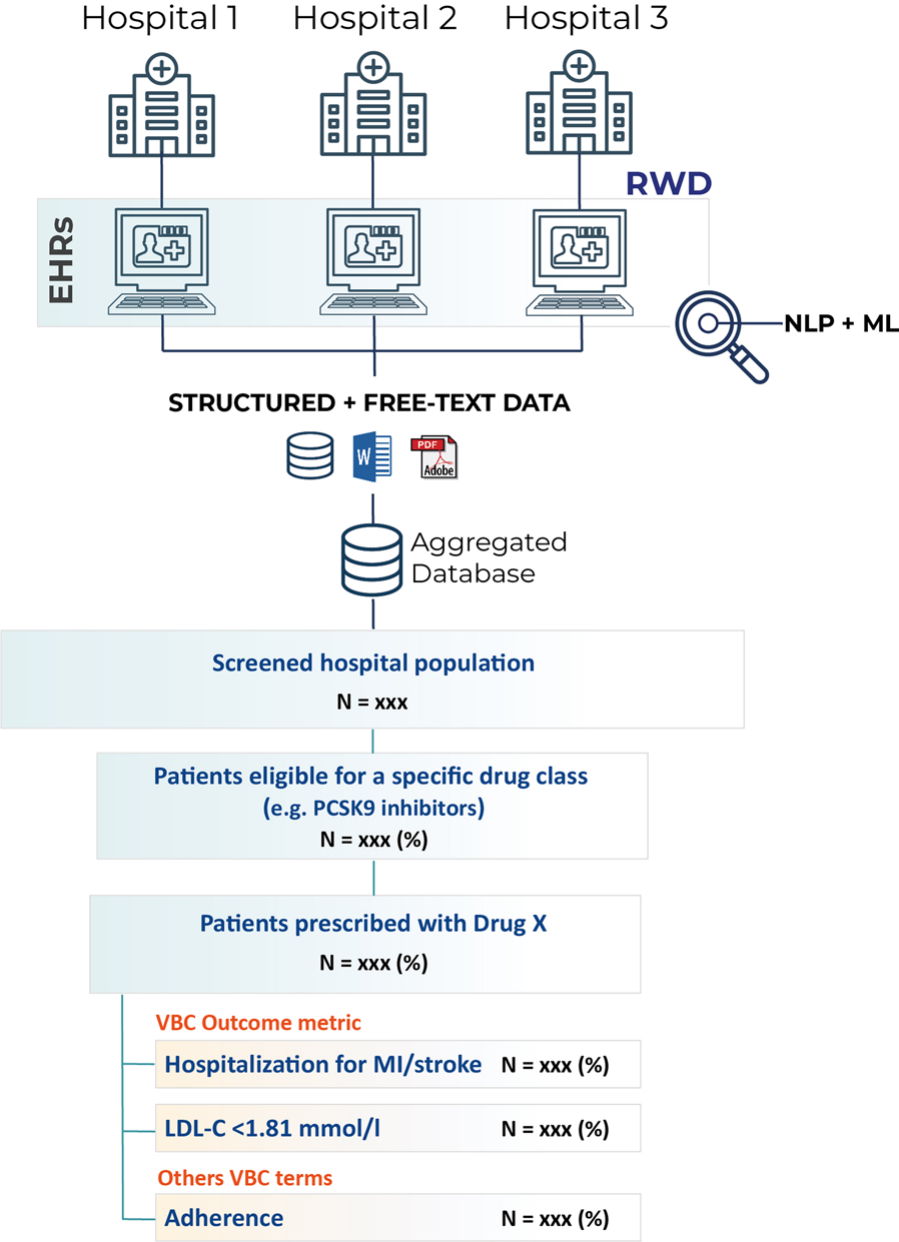
Using NLP and AI to guide VBC. Suggested methodology to analyze clinical outcomes in VBC using NLP. Data from single or multiple hospital sites are extracted and analyzed from anonymized EHRs using NLP. The structured and unstructured data are aggregated into a single database, where the target clinical population is screened. Patients using a specific drug (Drug X) can be extracted from all patients eligible for a specific pharmacological agent. Several clinical outcomes and metrics (as specified in the VBC) can be evaluated at the population level in those patients prescribed with the drug. This real-world information can be then used to adjust and negotiate pricing. EHR = electronic health record; LDL-C; low-density lipoprotein cholesterol; MI = myocardial infarction; ML = machine learning; NLP = Natural Language Processing; VBC = value-based contract

In multicentric NLP-based studies, the analysis of patient EHRs across healthcare centers provides accurate information regarding the performance of a given drug or its effectiveness against other competitors in the real world while ensuring a homogeneous data extraction in all hospitals involved. Furthermore, computerized NLP systems will cheapen the outcome evaluation process in VBCs from a resource use perspective. A hypothetical example using this methodological approach to evaluate the clinical outcomes of a given drug is shown in Fig. 1.

Tools and methods in AI and machine learning can be helpful not only in the implementation and development of VBCs, but also in their design [35]. AI can use big data to identify qualifying populations, select the appropriate pharmaceutical product and disease area, identify outcomes for measurement, and ultimately, assess the level of risk involved in the contract [35]. Finally, the involvement of a neutral third-party to manage the data could make the contracts more palatable to stakeholders involved in innovative contracting [36].

## Conclusions

NLP and related AI tools can positively contribute to VBC development due to their ability to extract and analyze real-time information to quantify treatment response in real-world settings, assess the financial impact of the treatment, and evaluate existing contracts by tracking outcomes over time.

## Supplementary Information

**Additional file 1. Supplemental Table 1.** List of approved and publicly available Value-Based Contracts (VBC) in USA, Europe, and UK (2015-2021).

## Data Availability

Data sharing is not applicable to this article as no data sets were generated or analyzed during the current study.
