# Supplementary material for: How can artificial intelligence optimize value-based contracting?
Source: J Pharm Policy Pract. 2022 Nov 18;15:85. doi: 10.1186/s40545-022-00475-3 (PMC9673444; doi:10.1186/s40545-022-00475-3)
Supplement: Supplementary file 1 — Additional file 1. Supplemental Table 1. List of approved and publicly available Value-Based Contracts (VBC) in USA, Europe, and UK (2015-2021). [file 40545_2022_475_MOESM1_ESM.pdf]

Supplemental Table 1. List of approved and publicly available Value-Based Contracts (VBC) in USA, Europe, and UK (2015-2021)

USA[1-3]						
**Brand name**	**Drug**	**Manufacturer**	**Payer(s)**	**Disease area**	**Outcome metric and terms**	**Year**
Stiolto®	tiotropium bromide/ olodaterol	Boehringer Ingelheim	Highmark	COPD	Reduces the total cost for both in-patient and out-patient COPD care for members vs. other available drugs	2019
Enbrel®	etanercept	Amgen	Abarca	Rheumatoid arthritis	Rebates to Abarca's clients for eligible members who discontinue the use of the drug after three months of treatment.	2019
Givlaari®	givosiran	Alnylam Pharmaceuticals	HPHC	Neurology Acute hepatic porphyria	Payers will pay the full value for GIVLAARI only when it delivers patient outcomes in the real-world setting similar to results demonstrated in clinical trials.	2019
Tecfidera®	dimethyl fumarate	Biogen	UPMC Health Plan	Multiple Sclerosis	Reimbursement by the drug manufacturer will be linked to reported measures of disability progression in a real-world population.	2019
Avonex®	interferon beta-1a	Biogen	UPMC Health Plan	Multiple Sclerosis	Reimbursement by the drug manufacturer will be linked to reported measures of disability progression in a real-world population.	2019
Luxturna®	voretigene neparvovecrzyl	Spark Therapeutics	Cigna	Retinal Dystrophy	Reduces net-cost to payer and determines payment amount based on selected clinical outcomes. If a Luxturna treatment fails (based on beneficiary light sensitivity), the payer would receive a rebate from Spark Therapeutics.	2019
Zolgensma®	onasemnogene abeparvovec-xioi	Novartis (Avexis)	Cigna; HPHC	Spinal muscular atrophy	Refunds will take place if drug is not effective over 5 years	2019
Mavyret®	glecaprevir/pibrentas vir	AbbVie	WSHCA	Hepatitis C	-	2019
Brilinta®	ticagrelor	AstraZeneca	UPMC Health Plan	Acute Coronary Events	Reimbursement linked to CV outcomes following a recent hospitalization for heart attack or unstable angina	2019
Invega Trinza®	paliperidone palmitate 2018	Janssen	Oklahoma Health Care Authority	Schizophrenia	Expenses for medical services	2018
Fycompa®	perampanel	Eisai	-	Epilepsy	Patients' outcomes and healthcare utilization and costs, including costs related to hospitalizations	2018
Stelara®	ustekinumab 2018 Immunology	Janssen	Prime Therapeutics	Psoriasis	Pharmacy and medical claims data to determine the duration of benefit from therapy	2018
Jardiance®	empagliflozin	Boehringer Ingelheim	UPMC Health Plan	Diabetes	Reimbursement linked to costs associated with clinical outcomes in the real-world population	2018
Orbativ®	oritavancin	Melinta Therapeutics	Oklahoma Health Care Authority	Acute Bacterial Skin Infections	Refunds if oritavancin results in a net increase in costs.<br>Outcome based on reduction in hospitalizations when compared with conventional therapies	2018
Aristada®	aripiprazole lauroxil	Alkermes	Oklahoma Health Care Authority	Schizophrenia	Patients 'adherence and improved outcomes	2018
Vivitrol®	naltrexone	Alkermes	UPMC Health	Opioid Dependence	Positive clinical outcomes; Reduction in the risk of relapse; Increase in adherence	2018
Cosentyx®	secukinumab	Novartis	Prime Therapeutics	Psoriasis	Medication persistence/costs	2018
Onpattro®	patisiran	Alnylam Pharmaceuticals	HPHC	hATTR Amyloidosis	Payment based on the ability to deliver outcomes in the real- world setting vs. performed clinical trials	2018
Jardiance®	empagliflozin	Boehringer Ingelheim	Highmark	Diabetes	analysis of the total cost of care for adults with type 2 diabetes and established cardiovascular disease	2018
Symbicort®	budesonide/formoter ol	AstraZeneca	HPHC	Asthma	Harvard Pilgrim to be charged a lower amount if asthma exacerbations/worsening of symptoms requiring medical intervention in real-world settings exceed thresholds determined in clinical trials.	2018

Symbicort®	budesonide/formoterol	AstraZeneca	Highmark	Asthma, COPD	If Symbicort doesn't perform as expected (based on outcomes published clinical trials), AstraZeneca will provide a corresponding rebate to Highmark.	2018
Victoza®	liraglutide	Novo Nordisk	SelectHealth	Diabetes	Effectiveness of Victoza to determine reimbursement to commercial clients if certain measures not achieved.	2018
Repatha®	evolocumab	Amgen	Abarca	Hypercholesterolemia	Amgen provides additional discounts if LDL cholesterol reductions not in line with clinical trial results.	2018
Jardiance®	empagliflozin	Boehringer Ingelheim	Prime Therapeutics	Diabetes	Medical and pharmacy claims data to evaluate and manage total cost of care	2018
Luxturna®	voretigene neparvovecrzyl	Spark Therapeutics	HPHC; Express Scripts	Retinal Dystrophy	If the therapy fails to perform as agreed, the payer will receive a rebate from Spark Therapeutics.	2018
Aimovig®	erenumab-aooe	Amgen / Novartis	Express Scripts	Migraine	Money-back guaranteed for plan sponsors when a patient discontinues therapy in the first 90 days.	2018
Victoza®	liraglutide	Novo Nordisk	Prime Therapeutics	Diabetes	Overall costs; effectiveness	2017
Xtampza ER®	oxycodone	Collegium	Cigna	Pain management	Collegium will reduce the cost of the medication if prescriptions exceed a specific daily dose threshold	2017
MS Portfolio	Multiple	Biogen	HPHC; Moda Care; Select Care	Multiple Sclerosis	Outcomes-based measures that tie pricing terms to an annualized relapse rate for their populations	2017
MS Portfolio	Multiple	Biogen	Prime Therapeutics	Multiple Sclerosis	Patients' adherence;Biogen provides an incremental discount based on patients who discontinue our therapies within the initial weeks of treatment.	2017
MS Portfolio	Multiple	Biogen	Abarca	Multiple Sclerosis	Biogen provides a rebate for discontinued treatment	2017
Brilinta®	ticagrelor	AstraZeneca	HPHC	Acute Coronary Events	Outcome based on the reduction in hospitalizations for acute coronary events in Brilinta-treated patients vs. other oral antiplatelets.	2017
Bydureon®	exenatide	AstraZeneca	HPHC	Diabetes	HbA1c levels and adherence get to a predetermined HbA1c goal	2017
Repatha®	evolocumab	Amgen	HPHC	Hypercholesterolemia	Full refund for the drug if a patient is hospitalized for MI or stroke after ≥6-month treatment with Repatha while adhering to the drug	2017
Enbrel®	etanercept	Amgen	HPHC	Rheumatoid arthritis	Two-year contract based on an effectiveness algorithm in real life. If patient scores are below a specified level while adhering to the drug, Harvard Pilgrim will pay less for Enbrel.	2017
Forteo®	teripararide	Eli Lilly	HPHC	Osteoporosis	Rewards improvement in adherence compared to baseline; if meaningful improvements are found, Lilly reduces the cost of the drug for Harvard Pilgrim	2017
Victoza®	liraglutide	Novo Nordisk	Humana	Diabetes	-	2017
Trulicity®	dulaglutide	Eli Lilly	HPHC	Diabetes	Price rebates tied to how well Trulicity performs vs. other GLP-1 agonists.	2016
Entresto ®	sacubitril/valsartan	Novartis	HPHC; Aetna; Cigna	Chronic heart failure	Novartis accepts lower net price if hospitalization reduction rates do not replicate clinical trials.	2016
Effient®	prasugrel	Eli Lilly	Humana	Acute coronary events	Links reimbursement to reductions in hospitalization rates	2016
Iressa®	getinib	Astra zeneca	Express Scripts	Lung cancer	Number of patients who fill the medicine three times	2016
Repatha®	evolocumab	Amgen	Cigna	Hypercholesterolemia	Amgen provides additional discounts if LDL cholesterol reductions not in line with clinical trial results.	2016
Brilinta®	ticagrelor	AstraZeneca	UPMC Health Plan	Acute Coronary Events	Measures the reduction in hospitalizations for repeat acute coronary events for patients on Brilinta against competitors	2016
Janumet®/Januvia®	Metformin/sitagliptin	Merk	Aetna	Diabetes	Merck will pay larger rebates if patients need to use additional therapy to reach HbA1C goals	2016
Repatha®	evolocumab	Amgen	HPHC	Hypercholesterolemia	Amgen provides additional discounts if LDL cholesterol reductions not in line with clinical trial results. Harvard Pilgrim can receive a full rebate if a patient experiences a MI or stroke while on the drug	2015
Repatha®	evolocumab	Amgen	CVS Health	Hypercholesterolemia	Net price linked to cholesterol reduction, appropriate patient use in return for preferred formulary status.	2015
Repatha®	evolocumab	Amgen	Prime Therapeutics	Hypercholesterolemia	Amgen provides additional discounts if LDL cholesterol reductions not in line with clinical trial results.	

Harvoni®	sofosbuvir/ledipasvir	Gilead	Cigna	Hepatitis C	Net price discount, linked to outcomes, in return for preferred formulary status.	2015
Praluent®	alirocumab	Sanofi, Regeneron	Cigna	Hypercholesterolemia	Extra price discounts if results are not in line with clinical trial results	2015
Avastin®	bevacizumab	Genentech	Priority Health	Lung Cancer	The shorter the PFS in a given patient, the greater the rebate to Priority Health. If a patient remained PFS on bevacizumab >6 monts, Priority Health would not receive a rebate.	2015
Tarceva®	erlotinib	Genentech	Express Scripts	Pancreatic/Lung cancer	Specific details are not spelled, but price could depend on OS in different diseases (indication-based pricing)	2015
			**EU-Italy (2, 4)**			
**Brand name**	**Drug**	**Manufacturer**	**Payer(s)**	**Disease area**	**Outcome metric and terms**	**Year**
Zolgensma®	Onasemnogene abeparvovec	Novartis	SSN	SMA	Reimbursement in patients up to 13.5 kg in weight with a) clinical diagnosis and onset of symptoms in the first 6 months of life or b) genetic diagnosis. Reimbursement deal with checkpoints at 12, 24, 36, and 48 months	2021
Rubraca®	rucaparib	Clovis Oncology	SSN	Ovarian Cancer	Reimbursement in women with relapsed platinum-sensitive ovarian cancer	2019
Yescarta®	axicabtagene ciloleucel	Gilead Sciences	SSN	diffuse large B-cell lymphoma (DLBCL)	Payments in three installments at 180, 270, and 365 days post-treatment, subject to outcomes being achieved and sustained. Specifics not disclosed	2019
Kymriah®	tisagenlecleucel	Novartis	SSN	B-cell acute lymphoblastic leukaemia (ALL)	Payments in three installments: the first at time of treatment, the subsequent two at 6 and 12 months post. Specifics not disclosed	2019
Kymriah	tisagenlecleucel	Novartis	SSN	diffuse large B-cell lymphoma (DLBCL)	Payments in three installments: the first at time of treatment, the subsequent two at 6 and 12 months post. Specifics not disclosed	2019
Holoclar®	ex vivo expanded autologous human corneal epithelial cells containing stem cells	Holostem Terapie Avanzate	SSN	Limbal stem cell deficiency (LSCD)	-	2017
Vargatef®	nintedanib	Boehringer Ingelheim	SSN	Non-small cell lung carcinoma	-	2017
Strimvelis®	autologous CD34+ enriched cell fraction	GSK	SSN	Severe combined immunodeficiency due to adenosine deaminase deficiency (ADA-SCID)	Pay-back in case of treatment failure	2016
Zelboraf®	vemurafenib	Roche	SSN	Melanoma	-	2015
Sustent®	Sunitinib	Pzifer	SSN	Pancreatic neuroendocrine tumors	-	2015
Abraxane®	paclitaxel-albumina	Briston Myers Squibb	SSN	Pancreatic tumor	Effectiveness of the two agents in the enhancement of anti-tumor response vs. either agent alone	2015
			**EU-SPAIN(5)**			
**Brand name**	**Drug**	**Manufacturer**	**Payer(s)**	**Disease area**	**Outcome metric and terms**	**Year**
Crysvita®	Burosumab	Kyowa Kirin	NHS	X-Linked hypophosphatemic rickets	Normalization of phosphate levels, resolving skeletal abnormalities and derived symptoms (pain). Follow up on the possible appearance of complications	2021
Dupixent®	Dupilumab	Sanofi-Aventis	NHS	Refractory atopic dermatitis	Reduction of symptoms (pruritus and dermatitis, prevention of exacerbations and minimization the risk of immunosuppressive treatment	2020
Veklury®	Remdesivir	Gilead Sciences	NHS	COVID-19	Prevention of the progression of the severity of the disease, promotion of clinical recovery and reduction of the length of hospital stay, and HCRU	2020

Kymriah®	tisagenlecleucel	Novartis	NHS	ALL; DLBCL	Complete response with negative MRD for over 18 months	2019
**UK[(6)]**						
**Brand name/Trial**	**Drug**	**Manufacturer**	**Payer(s)**	**Disease area**	**Outcome metric and terms**	**Year**
SPR1NT trial [NCT03505099]	Onasemnogene abeparvovec	Novartis	NICE/NHS	SMA	Clinical and cost-effectiveness	2021
DESTINY-Breast02 trial [NCT03523585]	Trastuzumab deruxtecan	Daiichi Sankyo	NICE/NHS	Unresectable or metastatic breast cancer	Relative effectiveness vs. other conventional therapies. PFS and OS data	2021
SOLO-2 trial	Olaparib	AstraZeneca	NICE/NHS	Advanced ovarian, fallopian tube or primary peritoneal cancer	PFS and OS data	2021
ZUMA-2 trial [NCT02601313]	Autologous anti-CD19-transduced CD3+ cells	Gilead	NICE/NHS	refractory mantle cell lymphoma	PFS, PPFS, and OS data and quality of life in long-term survivors	2021
ENGOT-OV16/NOVA trial	Niraparib	Glaxo Smith Kline	NICE/NHS	Advanced ovarian, fallopian tube and peritoneal cancer	OS and time to second progression; long-term PFS	2021
CLL13 trial (MO29596) NCT02950051]	Venetoclax/obinutuzumab	AbbVie	NICE/NHS	Chronic lymphocytic leukaemia	OS; Relative effectiveness vs. other conventional treatments	2020
ICARIA-MM trial [NCT02990338]	Isatuximab / pomalidomide / dexamethasone	Sanofi	NICE/NHS	relapsed and refractory multiple myeloma	PFS and OS data; estimation of time-on-treatment; Proportion of patients who progress onto 4th line therapy	2020
JAVELIN Renal 101 [NCT02684006]	Avelumab / axitinib	Merk / Pfizer	NICE/NHS	Untreated advanced or metastatic renal cell carcinoma	OS data; effectiveness for non-clear-cell disease; cost/benefits	2020
STARTRK-2 and STARTRK-NG trial	Entrectinib	Roche	-	Solid tumors	Prevalence and characterization of NTRK gene fusions and tumor type; long-term efficacy and safety; comparative effectiveness	2020
NAVIGATE [NCT02576431], SCOUT [NCT02637687] and LOXO-TRK-14001trial [NCT02122913]	Larotrectinib	Bayer	-	Solid tumors	Prevalence and characterization of NTRK gene fusions and tumor type; Prognostic importance of NTRK gene fusions; Management of patients; Long-term survival; Post-progression health utility state	2020
ENGOT-OV16/NOVA trial	Niraparib	Glaxo Smith Kline	NICE/NHS	Ovarian cancer	PFS and OS data	2019
Not specified	Cerliponase alfa	BioMarin	NICE/NHS	CLN2 Disease	Long-term disease stabilization; Improvements in motor language score starting distribution	2019
ARIEL3 trial	Rucaparib	Clovis oncology	NICE/NHS	Relapsed ovarian, fallopian tube or primary peritoneal cancer	OS data	2019
KEYNOTE-407 trial [NCT02775435]	Pembrolizumab / carboplatin / paclitaxel or nab-paclitaxel	Merk	NICE/NHS	metastatic squamous non-small-cell lung cancer	OS vs. standard care	2019
SOLO-1 trial	Olaparib	AstraZeneca	NICE/NHS	Advanced ovarian, fallopian tube or primary peritoneal cancer	Long-term OS; Proportion of patients who have residual disease at 2 years	2019
CheckMate 214 trial [NCT02231749]	Nivolumab / ipilimumab	Bristol-Myers Squibb	NICE/NHS	Advanced renal cell carcinoma	Long-term benefit in OS; Treatment duration; Subsequent therapies used in clinical practice; Proportion of patients with poor-risk or intermediate-risk renal cell carcinoma in clinical practice	2019

MONARCH 2 trial	Abemaciclib / fulvestrant	Eli Lilly	NICE/NHS	Breast cancer	Long-term survival data; Estimation of time-on-treatment	2019
PACIFIC trial	Durvalumab	Astra Zeneca	NICE/NHS	Unresectable non-small cell lung cancer	Long-term PFS and OS benefit following discontinuation	2019
JULIET trial	Tisangenlecleucel	Novartis	NICE/NHS	relapsed or refractory diffuse large B-cell lymphoma	OS data; proportion of people who need treatment for B-cell aplasia with Ig and duration of treatment	2019
ZUMA-1 trial [NCT02348216]	Axicabtagene ciloleucel	Gilead Sciences	NICE/NHS	diffuse large B-cell lymphoma and primary mediastinal B-cell lymphoma	OS at 5-year follow-up; convergence of PFS and OS curves at 5-years follow-up; intravenous Ig use	2019
EMPOWER-CSCC 1 trial	Cemiplimab	Sanofi	NICE/NHS	cutaneous squamous cell carcinoma	Baseline characteristics of the target population<br>Long-term benefit<br>Comparative effectiveness with standard treatments in clinical practice	2019
CASTOR trial	Daratumumab / bortezomib / dexamethasone	Janssen	NICE/NHS	multiple myeloma	OS at 6 years	2019
KEYNOTE-087 trial	Pembrolizumab	Merck	NICE/NHS	refractory classical Hodgkin lymphoma	The time at which allogeneic stem cell transplant occurs. proportion of patients who receive an allogeneic stem cell transplant	2018
Not specified	Daratumumab	Janssen	NICE/NHS	multiple myeloma	OS<br>Subsequent treatment following daratumumab	2018
PROFILE 1001 trial	Crizotinib	Pfizer	NICE/NHS	ROS1-positive advanced non-small-cell lung cancer	Effectiveness comparation with standard care<br>Post-progression and overall survival estimates	2018
IMvigor 130 trial	Atezolizumab	Roche	NICE/NHS	Untreated metastatic urothelial cancer	OS and PFS compared with standard care<br>HRQoL for patients with SD and PD receiving atezolizumab and standard care	2018
ELIANA, ENSIGN and B2101J trials	Tisangenlecleucel	Novartis	NICE/NHS	relapsed or refractory B-cell acute lymphoblastic leukaemia	Curative nature of tisangenlecleucel<br>Subsequent stem cell transplant<br>Number of people who need treatment for B-cell aplasia with intravenous Ig, and duration of the treatment	2018
KEYNOTE-054 trial [NCT02362594]	Pembrolizumab	Merk<br>Sharp<br>Dohme	NICE/NHS	melanoma	Distant metastases free survival and OS benefit vs, standard therapy	2018
TOURMALINE MM-1 trial	Ixazomib / lenalidomide / dexamethasone	Takeda	NICE/NHS	relapsed or refractory multiple myeloma	OS; Duration of treatment; Quality of life (EQ 5D)	2018
Study 1118E and the phase 3 Study 1127 [iNNOVATE	Ibrutinib	Janssen	NICE/NHS	Waldenstrom's macroglobulinaemia	Pre-progression mortality and treatment duration	2017
CheckMate 141	Nivolumab	Bristol-Myers Squibb	NICE/NHS	Recurrent or metastatic squamous-cell carcinoma of the head and neck	Effectiveness according to PD-L1 expression	2017
Not specified	Venetoclax	AbbVie	NICE/NHS	chronic lymphocytic leukaemia	OS; PPS	2017

HPHC = Havard Pilgrim Health Care; OS = Overall survival; PFS = Progression-free survival; PPFS = Post-progression-free survival; SSN = National Health System (Italy); UPMC = University of Pittsburgh Medical Center; WSHCA = Washington State Healthcare Authority.

SUPPLEMENTAL REFERENCES

1. PHRMA.ORG. Innovative contracts: 2009-2020. 2020.
2. Jørgensen J, Kefalas P. The use of innovative payment mechanisms for gene therapies in Europe and the USA. Regenerative Medicine. 2021;16(4):405-22.
3. Group DR. Value-Based Contracting in Pharmaceuticals - January 2019 update. 2019.
4. AIFA. Lista aggiornata dei Registri e dei Piani Terapeutici 2021.
5. Government MoHotS. istema de Información para determinar el Valor Terapéutico en la Práctica Clínica Real de los Medicamentos de Alto Impacto Sanitario y Económico en el SNS (VALTERMED). 2021.
6. (UK) NIfHaCE. Updated list of approved drugs. 2021.
